# A comparative analysis of lip print patterns for sex determination and permanency using lipstick, latent and digital methods in North Bihar

**DOI:** 10.6026/973206300213936

**Published:** 2025-10-31

**Authors:** Sumanta Kumar Kolay, Rohit Sharma, Ajit Kumar Chaudhary, Sunil Sharma, Keerti Bhardwaj, Neetha Bhargava

**Affiliations:** 1 Department of Oral and Maxillofacial Pathology & Microbiology, Nims Dental College & Hospital, Nims University Rajasthan, Jaipur, India; 2 Department of Pathology, Madhubani Medical College and Hospital, Madhubani, Bihar, India; 3 Department of Oral and Maxillofacial Surgery, Nims Dental College & Hospital, Nims University Rajasthan, Jaipur, India; 4 Department of Anatomy, Jaipur National University Institute for Medical Sciences, Jaipur, Rajasthan, India; 5 Department of Periodontology, Nims Dental College & Hospital, Nims University Rajasthan, Jaipur, India

**Keywords:** Cheiloscopy, lip prints, sex determination, forensic identification, permanency, Suzuki classification

## Abstract

Forensic identification often relies on fingerprints, but their absence necessitates alternative tools such as lip prints (cheiloscopy). Therefore, it is of interest to evaluate the permanency of lip prints over one year and compared three recording methods-lipstick, latent prints and digital photography-for sex determination in the North Bihar population. Hence participants were assessed at baseline, 6 months and 12 months using Suzuki and Tsuchihashi classification. Lip print patterns remained stable over time with no significant intra- or inter-method differences and sex determination accuracy ranged from 63.8% to 67.2% across methods. Thus, we show lip prints are permanent and recording methods equally effective, though accuracy in sex determination requires further enhancement for forensic reliability.

## Background:

Forensic science plays a pivotal role in the justice system, providing critical evidence for individual identification in both criminal investigations and mass disaster scenarios [1]. Among various biometric markers, fingerprints have long been considered the gold standard for personal identification due to their uniqueness and permanence [2]. However, in cases where fingerprints are unavailable, compromised, or intentionally obscured, alternative identification methods become essential [3]. Lip prints, the study of which is known as cheiloscopy, have gained attention as a potential complementary forensic tool [4]. The characteristic patterns of grooves and wrinkles on the labial mucosa are believed to be unique to each individual, even among identical twins [5]. These patterns develop during the sixth week of intrauterine life and are thought to remain unchanged throughout life, unaffected by various pathological conditions [6]. The potential of lip prints as forensic evidence was first recognized in the early 20th century, with anthropologist R. Fischer describing the biological occurrence of furrows on human lips in 1902 [7]. Later, in 1932, French criminologist Edmond Locard recommended the utility of these patterns for individual identification [8]. The forensic application of lip prints requires addressing two critical aspects: their permanence over time and the reliability of different recording methods [9]. Several classification systems have been proposed to categorize lip print patterns, with the Suzuki and Tsuchihashi classification being widely adopted [10]. This system categorizes lip prints into six types based on groove patterns, providing a standardized approach for analysis [11].

Recent studies have explored the utility of lip prints in sex determination, with varying degrees of success [12, 13-14]. The methods for recording lip prints have evolved significantly, with three primary techniques currently in use: the lipstick method, latent lip print method and digital photography [15]. Each method presents distinct advantages and limitations. The lipstick method involves applying lipstick to the lips and transferring the print to a substrate, offering simplicity but potential for distortion [16]. The latent lip print method, similar to fingerprint dusting, involves developing invisible lip prints using powders or chemicals [17]. Digital photography provides a non-invasive approach, allowing for enhancement and detailed analysis [18]. Despite these advancements, significant gaps remain in our understanding of lip print permanence over extended periods and the comparative efficacy of different recording methods for sex determination [19]. Most studies have been conducted on limited populations, with minimal research focusing on the North Bihar population of India [20]. Furthermore, the long-term stability of lip prints beyond six months has not been adequately investigated [21]. Therefore, it is of interest to address these gaps by evaluating the permanency of lip prints over a one-year period and comparing the efficacy of three recording methods-lipstick, latent lip print and digital photography-for sex determination in the North Bihar population.

## Materials and Methods:

## Study design and setting:

An observational cross-sectional study with comparative analysis was conducted in the Department of Oral Pathology and Microbiology, NIMS Dental College and Hospital, Jaipur, over a 12-month period. The study received approval from the institutional ethics committee (Reference No. NIMS/IEC/2021/045).

## Study population and sampling:

Participants were recruited from the North Bihar population through convenience sampling. Inclusion criteria comprised individuals aged 10-60 years with healthy labial mucosa and complete dentition. Exclusion criteria included individuals with diabetes insipidus, diabetes mellitus, or other systemic diseases causing excessive dehydration of lip mucosa; those with inflamed oral mucosa, pathologies, lip deformities (e.g., cleft lip or lip pits), or physical/chemical injuries of the lips; and individuals with known allergic reactions to lipstick or cellophane tape.

## Informed consent:

Written informed consent was obtained from all participants (or guardians for minors) after explaining the study procedure in their preferred language (English or Hindi). All participants were assured of confidentiality and their right to withdraw from the study at any time without consequences.

## Procedure:

## Lip print recording:

All participants underwent lip print recording using three methods at baseline (T1), 6 months (T2) and 12 months (T3). The procedures were standardized as follows:

## Latent lip print method:

[1] Lips were cleaned with moist gauze and patted dry.

[2] Participants were instructed to gently press their lips against a glass slab for 3-4 seconds.

[3] The glass slab was dusted with black fingerprint powder.

[4] Excess powder was removed and the print was transferred to a white bond sheet using cellophane tape.

## Lipstick method:

[1] A thin layer of dark-colored lipstick was uniformly applied to the lips using a disposable applicator.

[2] Participants were asked to rub their lips gently for even distribution.

[3] Participants pressed their lips onto the sticky side of cellophane tape.

[4] The tape with the lip print was transferred to a white bond sheet.

## Digital method:

[1] Participants were positioned standing erect with their head in the Frankfurt plane.

[2] Lip photographs were taken twice in natural light using a digital camera mounted on a tripod at a height of 5.5 feet.

[3] Images were saved on a computer for analysis.

## Lip print classification:

Lip prints were divided into four quadrants according to the dental formula and classified using the Suzuki and Tsuchihashi classification:

[1] Type I: Clear-cut vertical grooves across the entire lip

[2] Type I: Partial-length vertical grooves

[3] Type II: Branched grooves

[4] Type III: Intersecting grooves

[5] Type IV: Reticular grooves

[6] Type V: Other patterns

[7] Non-traceable (NT): No discernible pattern

For statistical analysis, types were numbered from 0 to 6, with 0 representing non-traceable prints and 1-6 representing Types I-V respectively.

## Statistical analysis:

Data were analyzed using SPSS software (Version 25.0, IBM Corp., USA). Descriptive statistics were presented as mean ± standard deviation (SD) for continuous variables and frequencies (percentages) for categorical variables. One-way ANOVA was used to compare lip print recordings at different time points within each method. Tukey's ANOVA was employed for pairwise comparisons between time points. Inter-method comparisons were performed using ANOVA and post-hoc tests. A p-value <0.05 was considered statistically significant.

## Results:

All participants completed the three follow-up assessments at baseline (T1), 6 months (T2) and 12 months (T3). No statistically significant differences were observed in lip print patterns recorded at T1, T2 and T3 using any of the three methods in both males and females (p>0.05). Table 1 (see PDF) presents the detailed analysis of lip print recordings over time. Pairwise comparisons between time points using Tukey's ANOVA revealed no statistically significant differences (p>0.05) for any method in either gender, confirming the stability of lip print patterns over the 12-month period. Inter-method comparisons showed no significant differences in efficacy at T2 and T3 (p>0.05). However, at T1, significant differences were observed between the latent lip print method and the other two methods in males (p=0.0148). Table 2 (see PDF) presents the comparison of lip print recordings by the three methods at each time point. The accuracy of sex determination using lip print patterns varied by method and time point. Overall, the lipstick method showed the highest accuracy (67.2%), followed by the digital method (65.5%) and the latent lip print method (63.8%). Table 3 (see PDF) presents the sex determination accuracy for each method at different time points. The most common lip print pattern in males was Type II (branched grooves), observed in 32.8% of cases, while females predominantly showed Type I (vertical grooves) in 36.2% of cases. However, these patterns were not sufficiently distinctive to achieve high accuracy in sex determination.

## Discussion:

This study investigated the permanence of lip prints over a one-year period and compared the efficacy of three recording methods for sex determination in the North Bihar population. The findings demonstrate that lip prints remain stable over time and that all three recording methods show comparable efficacy, supporting their potential application in forensic identification. The stability of lip prints observed in our study aligns with previous research suggesting that lip patterns remain unchanged over time [22]. Abdel and Nagasupriya reported no changes in lip print patterns over a three-month period using the lipstick method [23, 24]. Similarly, our study extends these findings by demonstrating stability over a full year using three different recording methods, strengthening the evidence for lip print permanence. The comparable efficacy of the three recording methods observed in our study, particularly at T2 and T3, is noteworthy. While some previous studies have suggested the superiority of digital methods [25], our findings indicate that with proper technique, all three methods can yield reliable results. The minor variations observed at T1 between the latent lip print method and the other methods in males may be attributed to the learning curve associated with the latent technique, which requires careful powder application and transfer [26]. As the study progressed, these differences diminished, suggesting that operator experience plays a crucial role in the reliability of lip print recording. The sex determination accuracy rates observed in our study (63.8-67.2%) are modest compared to some previous reports [27]. This discrepancy may be attributed to population-specific variations, differences in classification systems, or methodological approaches. The predominance of Type II patterns in males and Type I patterns in females observed in our study partially aligns with previous research. Several studies have reported that vertical groove patterns (Type I) are more common in females, while branched patterns (Type II) are more frequent in males [3]. However, the overlap in pattern distribution between genders limits the reliability of lip prints for sex determination in isolation. Thus utility of lip print analysis in forensic science and emphasize the need for further research to enhance the interpretation of lip print evidence [28].

## Conclusion:

Lip prints were found to be stable over one year, confirming their permanence as a biometric marker. All three recording methods demonstrated comparable efficacy, though sex determination accuracy remained modest. Cheiloscopy thus holds promise as a complementary forensic tool, warranting further research with larger samples and advanced techniques.
